# Crystal structure, Hirshfeld surface analysis, DFT and mol­ecular docking studies of ethyl 5-amino-2-bromo­isonicotinate

**DOI:** 10.1107/S2056989024010594

**Published:** 2024-11-08

**Authors:** Harish Kumar Mahadevaiah, Harishkumar Shivanna, Anil Kumar Hanumaiah, Devarajegowda Hirehalli Chikkegowda, Palakshamurthy Bandrehalli Siddagangaiah

**Affiliations:** a Department of Physics Yuvaraja's College University of Mysore,Mysore 570005 Karnataka India; b Department of Pharmaceutical Chemistry Kuvempu University Shimoga Karnataka 577451 India; c Department of Physics, Government First Grade College, Chikkabalapura, Karnataka-562101, India; d Department of PG Studies and Research in Physics Albert Einstein Block UCS Tumkur University, Tumkur Karnataka 572103 India; Harvard University, USA

**Keywords:** crystal structure, Hirshfeld surface, DFT studies, mol­ecular docking, isonicotinate

## Abstract

In the title compound, C_8_H_9_BrN_2_O_2_, the C—O—C—C torsion angle between isonicotine and the ethyl group is 180.0 (2)°. Intra­molecular N—H⋯O and C—H⋯O inter­actions consolidate the mol­ecular structure. In the crystal, N—H⋯N inter­action form *S*(5) zigzag chains along [010]. The most significant contributions to the Hirshfeld surface arise from H⋯H (33.2%), Br⋯H/H⋯Br (20.9%), O⋯H/H⋯O (11.2%), C⋯H/H⋯C (11.1%) and N⋯H/H⋯N (10%) contacts. The topology of the three-dimensional energy frameworks was generated using the B3LYP/6–31 G(d,p) model to calculate the total inter­action energy. The net inter­action energies for the title compound are *E*_ele_ = 59.2 kJ mol^−1^, *E*_pol_ = 15.5 kJ mol^−1^, *E*_dis_ = 140.3 kJ mol^−1^ and *E*_rep_ = 107.2 kJ mol^−1^ with a total inter­action energy *E*_tot_ of 128.8 kJ mol^−1^. The mol­ecular structure was optimized by density functional theory (DFT) at the B3LYP/6–311+G(d,p) level and the theoretical and experimentally obtained parameters were compared. The frontier mol­ecular orbitals HOMO and LUMO were generated, giving an energy gap Δ*E* of 4.0931 eV. The MEP was generated to identify active sites in the mol­ecule and mol­ecular docking studies carried out with the title compound (ligand) and the covid-19 main protease PDB ID: 6LU7, revealing a moderate binding affinity of −5.4 kcal mol^−1^.

## Abstract 2

Theoretical and experimental structural studies of the title compound were undertaken using X-ray and DFT methods. The inter­actions present in the crystal were analyzed using Hirshfeld surface and MEP surface analysis. Docking studies with a covid-19 main protease (PDB ID: 6LU7) as the target receptor indicate that the synthesized compound may be a potential candidate for pharmaceutical applications.

## 1. Chemical context

The derivatives of isonicotinate are enanti­omerically enriched in the *R* and *S* configuration. The mol­ecules associated with 2-methyl­alkyl isonicotinate and nicotinate exhibit *R* and *S* configurations at the mol­ecular level. These compounds demonstrate a good anti-fungal activity against different phytopathogenic fungi species and they play significant role in the reduction of the damage at the plant cell and chloro­plast levels (Huras *et al.*, 2017 ▸). Isonicotinate ligands with an organoruthenium(II) ion form organometallic complexes that exhibit anti-cancer activities (Liu *et al.*, 2012 ▸). Silver complexes with nicotinate-based ligands exhibit anti-bacterial activity against clinically isolated pathogens (Abu-Youssef *et al.*, 2007 ▸). Various metal complexes with nicotinate moieties have been used to develop phytopathogenic drugs. Most importantly, organotin isonicotinate derivatives are extensively used in the development of anti­proliferative drugs, which play a significant role at the innermost layer of cells lining blood vessels and lymphatic vessels (Vieriu *et al.*, 2021 ▸). These drugs are used in drug-eluting stents to inhibit vascular smooth muscle cell proliferation possesses, exhibit considerable vasodilator properties and are also used to boost endothelial protective properties (Girgis *et al.*, 2006 ▸). The isonicotinate-derived *meso-*tetra­aryl­porphyrin exhibits anti-oxidant, anti-fungal and allelopathic activities (Dardouri *et al.*, 2024 ▸). As part of our studies of this family of materials, we now present the synthesis, structure and Hirshfeld surface analysis of the title compound.
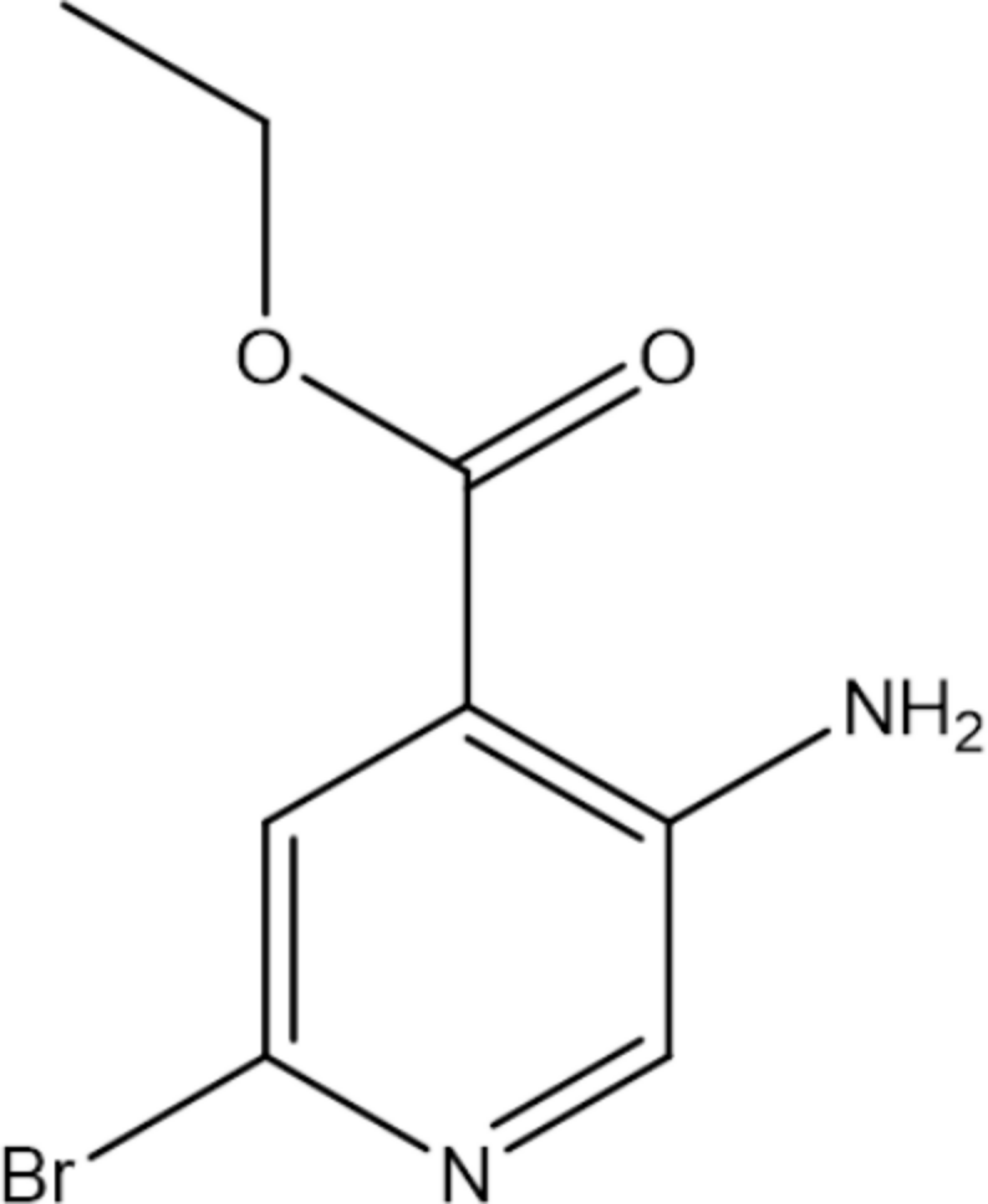


## 2. Structural commentary

**Figure 1:**
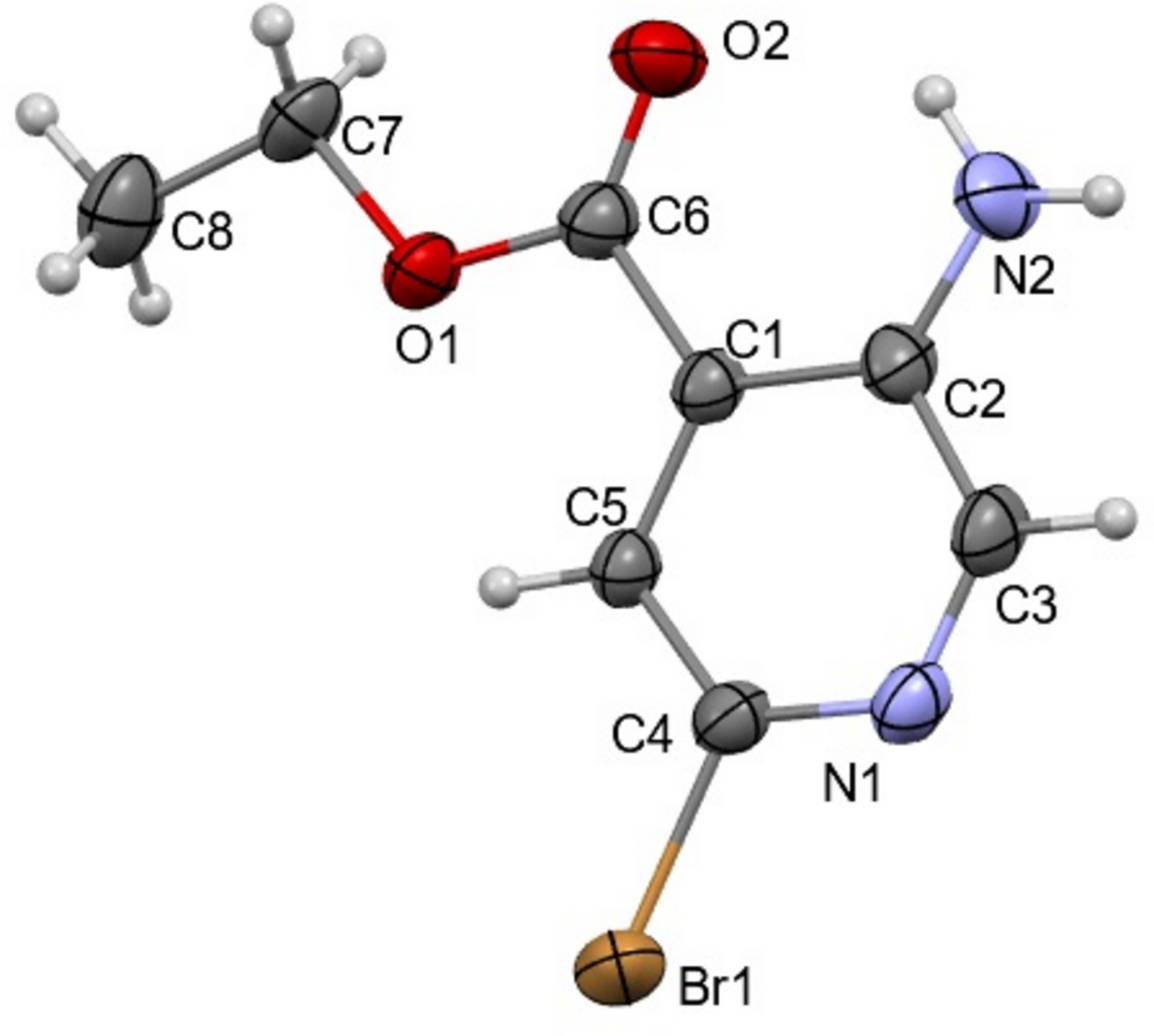
The title mol­ecule with the atom-labeling scheme and 50% probability displacement ellipsoids.

The mol­ecular structure of title compound, which crystallizes in the monoclinic space group *P*21/*c*, is shown in Fig. 1 ▸. The amino-2-bromo­isonicotinate ring system is essentially planar, with an r.m.s deviation of 0.043 (2) Å. The whole mol­ecule is essentially planar, the dihedral angle between the mean planes defined by the isonocotine moiety and the side chain being 4.30 (2)°. The C6—O1—C7—C8 torsion angle of 175.2 (2)° indicates that the ethyl group is in a planar orientation with the isonocotine ring [see also: C1—C6—O1—C7 = −178.0 (2)° and N2—C2–C1–C6 = −1.2 (4)°]. The mol­ecular structure is consolidated by N2—H2*B*⋯O2 and C5—H5⋯O1 intra­molecular inter­actions (Table 1 ▸).

## 3. Supra­molecular features

**Figure 2:**
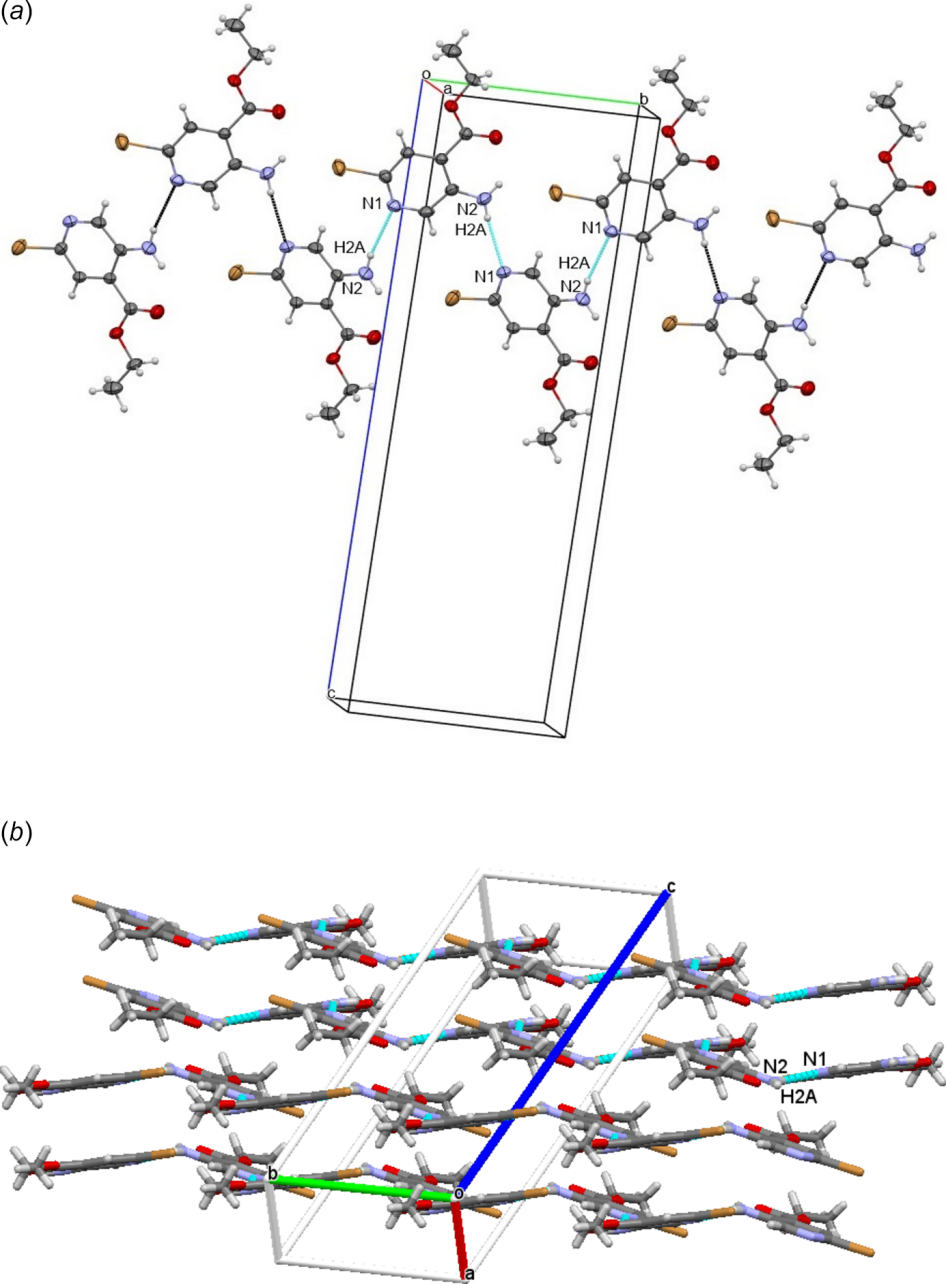
(*a*) The three-dimensional mol­ecular packing of the title compound. Dashed lines indicate N—H⋯N inter­molecular hydrogen bonds forming zigzag chains along [010]. (*b*) Perspective view of the mol­ecular sheets.

In the crystal, N2—H2*A*⋯N1 inter­actions (Table 1 ▸) link the mol­ecules into *S*(5) zigzag chains along [010] as shown in Fig. 2 ▸*a* and makes mol­ecular sheets through N—H⋯N interactions between the four independent molecules in the unit cell, as shown in Fig. 2 ▸*b*.

## 4. Database survey

A search of the Cambridge Structural Database (CSD, version 5.42, update of November 2020; Groom *et al.*, 2016 ▸) was undertaken for mol­ecules containing ethyl 3-amino­isonicotinate, 5-amino-2-bromo­isonicotinic acid and ethyl 2-bromo­isonicotinate fragments, but no hits were found. However, 29 hits were found in a search for mol­ecules containing an ethyl isonicotinate fragment. Among those, in the structures with CSD refcodes ROMMIQ (Wang *et al.*, 2009 ▸), SILPOT (Wan *et al.*, 2007 ▸), XEZDEM (Han *et al.*, 2007 ▸) and XIMBIF (Li *et al.*, 2007 ▸), the C—C—O—C torsion angles associated with the isonicotinate are 177.93 (2), −168.46 (3), −168.46 (3) and 176.82 (2)°, respectively, with the same *anti* conformation as in the title compound where the comparable torsion angle is 180.0 (2)°.

## 5. Hirshfeld surface analysis and inter­action energies

**Figure 3:**
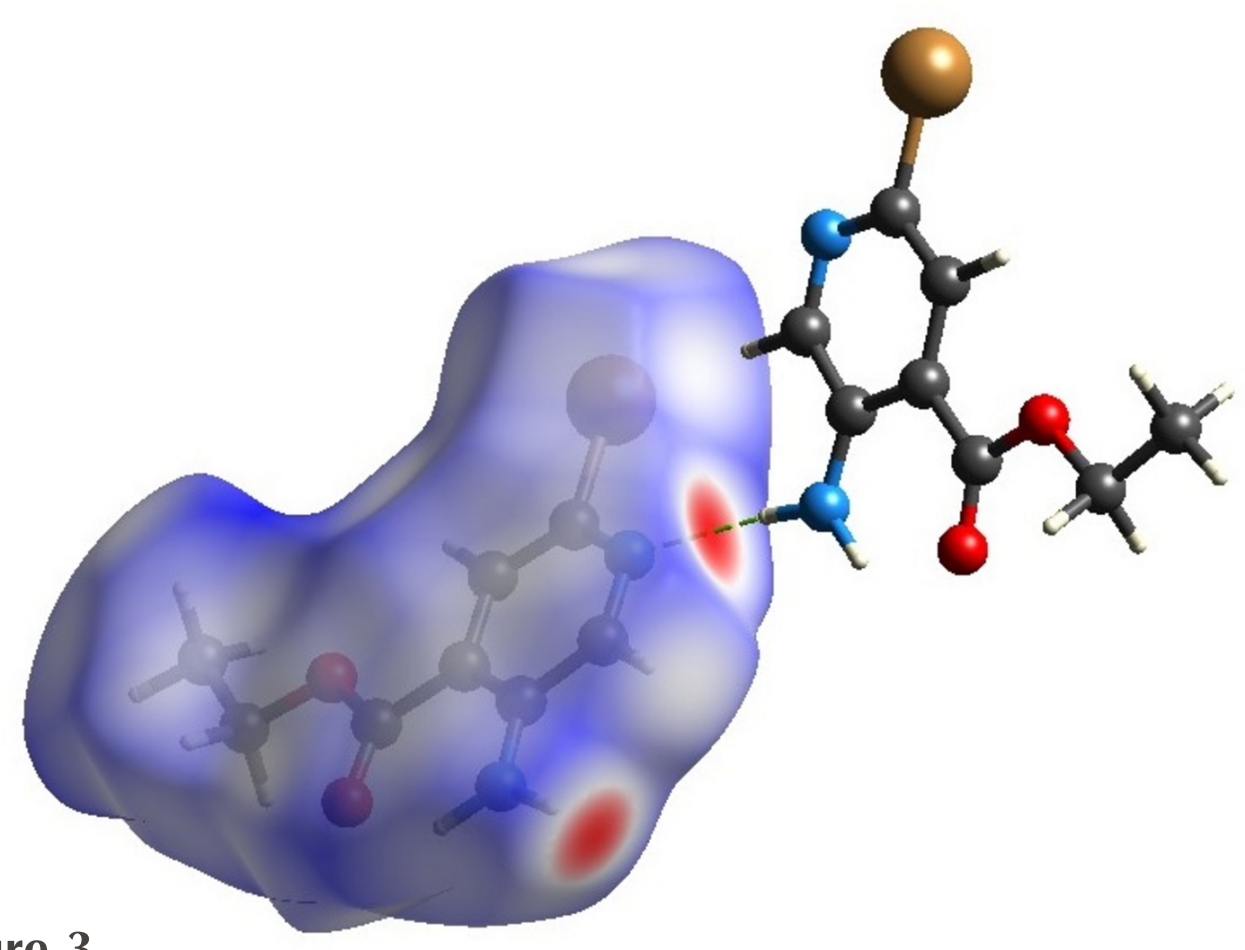
The Hirshfeld surface mapped over *d*_norm_ with red spots corresponding to the electronegative site of the nitro­gen of the mol­ecule.

**Figure 4:**
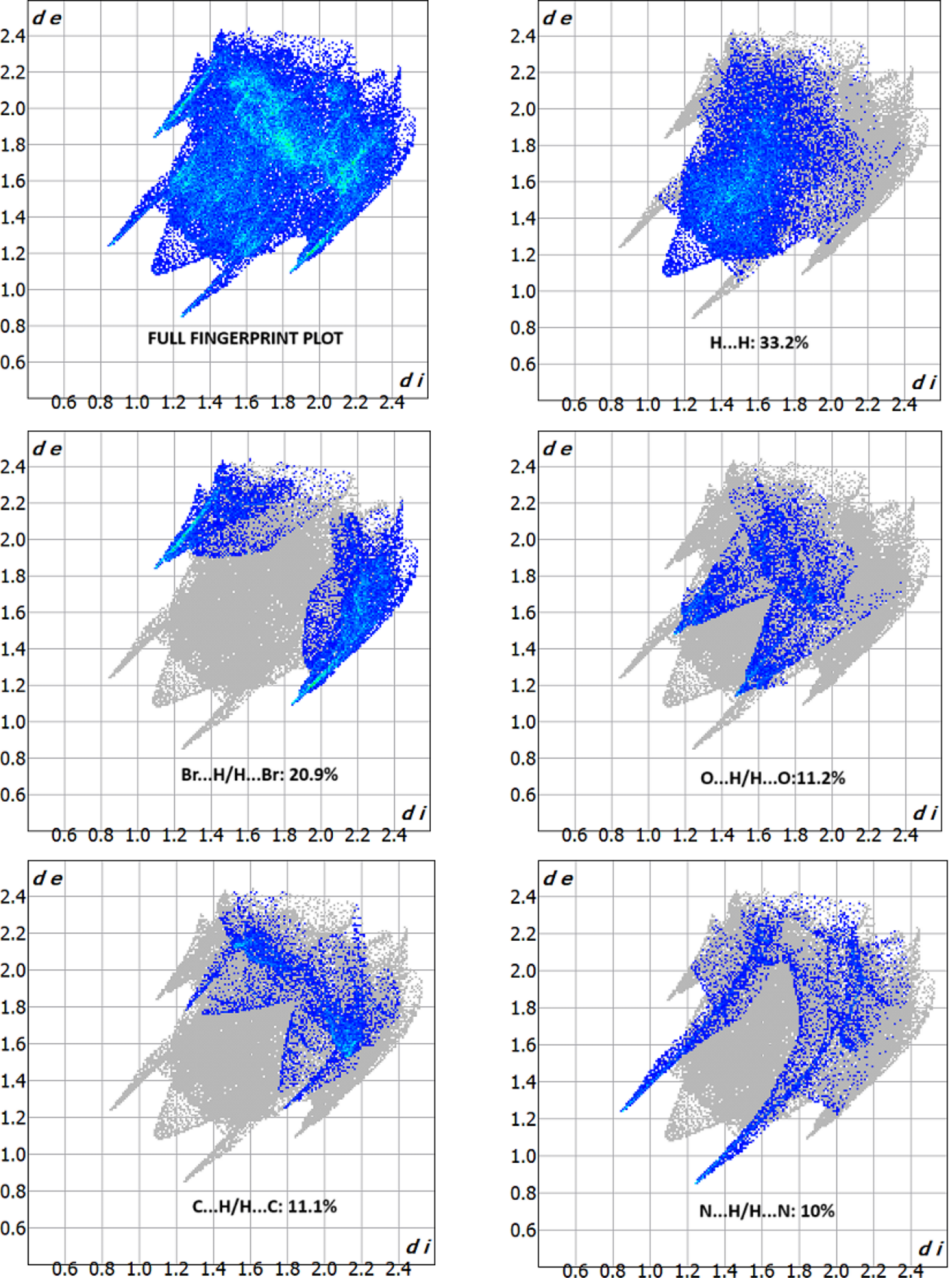
The fingerprint plots of the title mol­ecule, showing the major contributions to the Hirshfeld surface from H⋯H (33.2%), Br⋯H/H⋯Br (20.9%), O⋯H/H⋯O (11.2%), C⋯H/H⋯C (11.1%) and N⋯H/H⋯N (10%) contacts.

**Figure 5:**
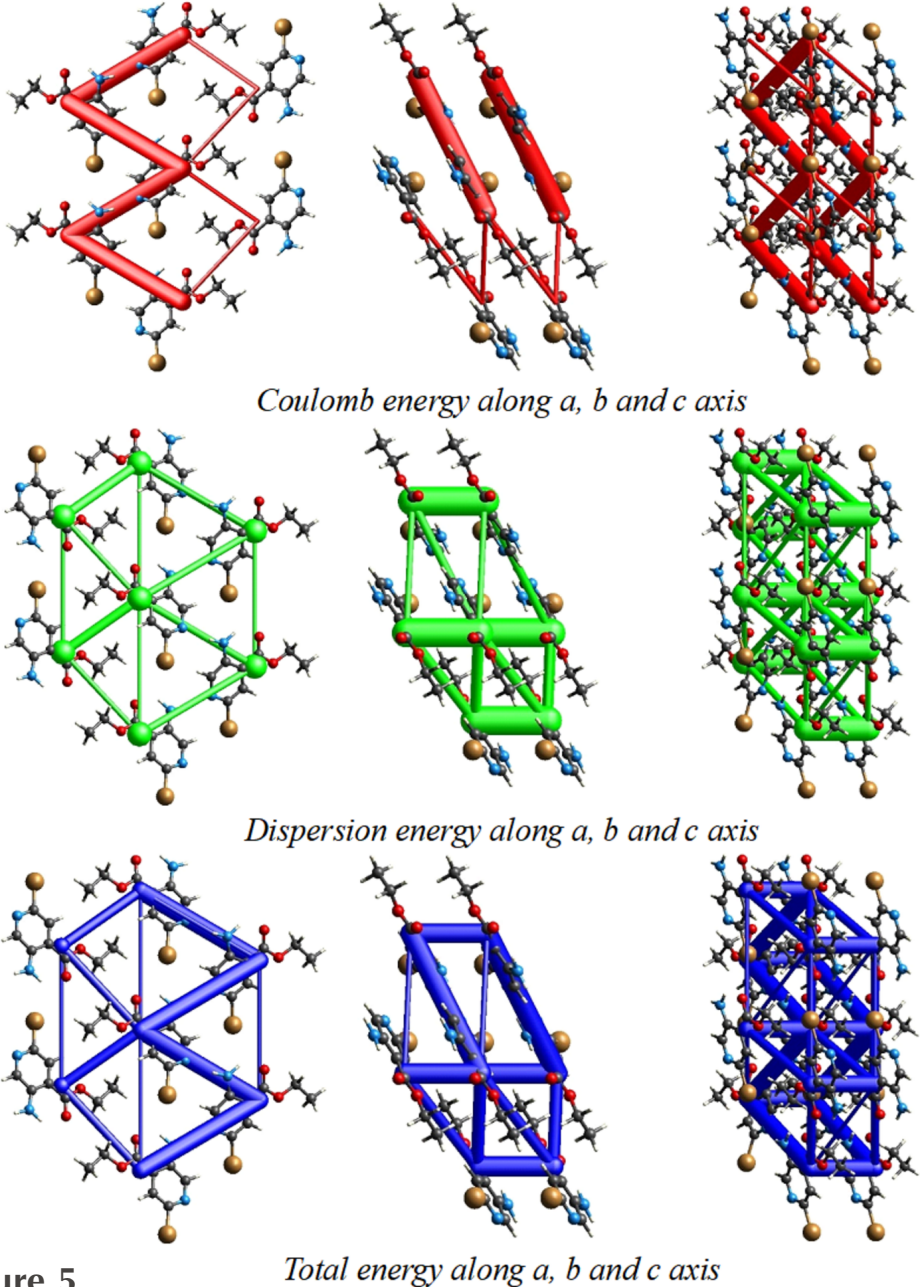
The topology of the energy frameworks along the *a*, *b* and *c* axes for inter­action energies.

*CrystalExplorer17.5* (Turner *et al.*, 2017 ▸) was used to perform a Hirshfeld surface analysis to qu­antify the various inter­molecular inter­actions. Fig. 3 ▸ illustrates the Hirshfeld surface mapped over *d*_norm_ with red spots corresponding to electronegative site of the nitro­gen through which a short contact N2—H2*A*⋯N1 forms a hydrogen-bonded chain. The fingerprint plots in Fig. 4 ▸ indicate that the major contributions to the Hirshfeld surface of the crystal structure are from H⋯H (33.2%), Br⋯H/H⋯Br (20.9%), O⋯H/H⋯O (11.2%), C⋯H/H⋯C (11.1%) and N⋯H/H⋯N (10%) contacts. The characteristic spikes in the N⋯H/H⋯N plot indicate the presence of the N2—H2*A*⋯N1 hydrogen bond listed in Table 1 ▸. The three-dimensional inter­action energy between the mol­ecules of the title compound were computed using the basis set B3LYP/6-31G(d,p). The net inter­action energies are *E*_ele_ = 59.2 kJ mol^−1^, *E*_pol_ = 15.5 kJ mol^−1^, *E*_dis_ = 140.3 kJ mol^−1^, *E*_rep_ = 107.2 kJ mol^−1^ with a total inter­action energy *E*_tot_ of128.8 kJ mol^−1^. The topology of the energy frameworks along the *a*, *b* and *c* axes for the different contributions (Coulombic energy, dispersion energy and total energy) are shown in Fig. 5 ▸.

## 6. DFT Studies

**Figure 6:**
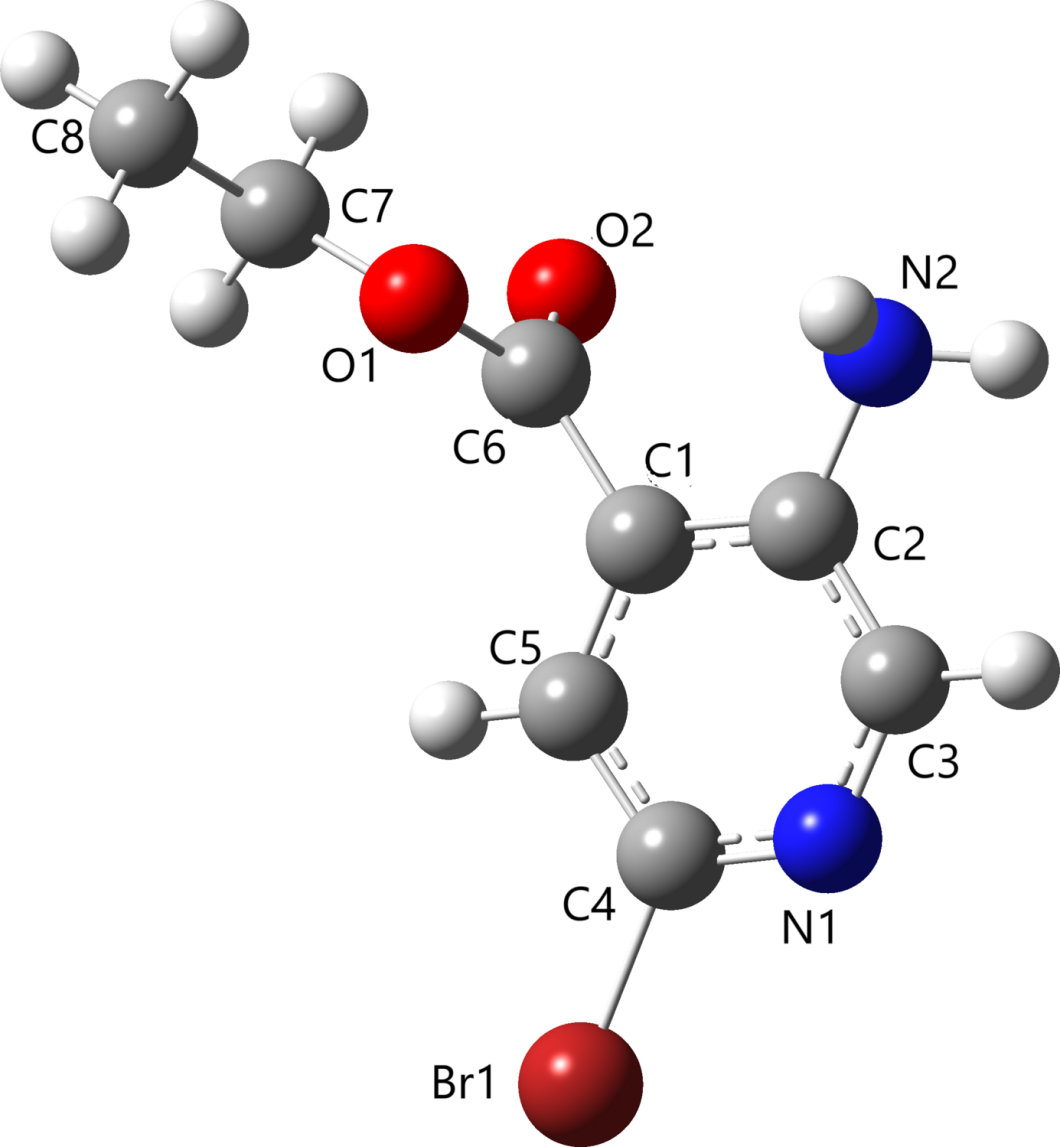
The optimized mol­ecular structure of the title compound generated using *Gaussian 09W*.

**Figure 7:**
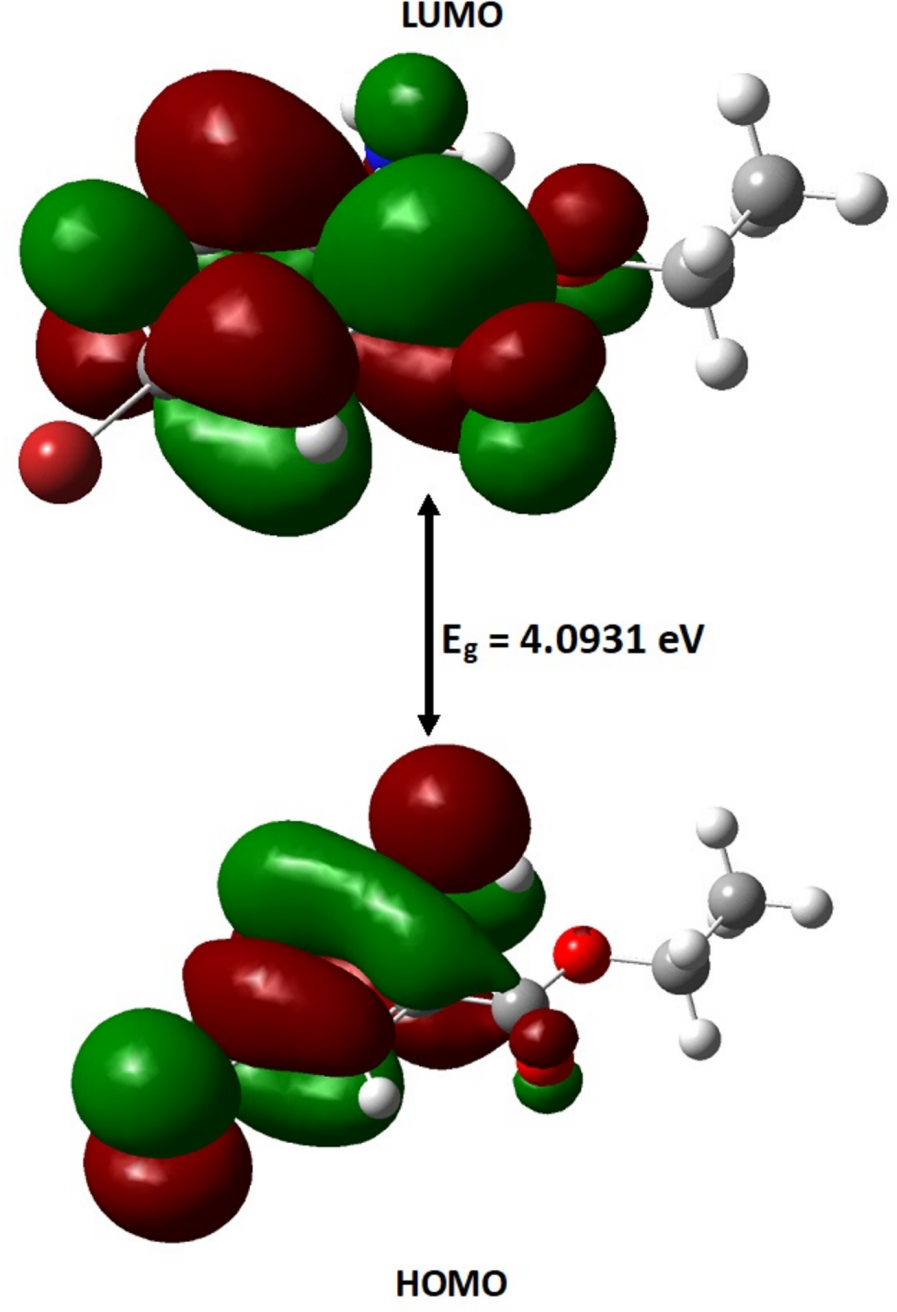
The frontier mol­ecular orbitals HOMO and LUMO energy levels, with the energy gap Δ*E* = 4.0931 eV.

The title compound was studied by DFT calculations in the gas phase at the B3LYP/6-311+G(d,p) level of theory with *Gaussian 09W* (Frisch *et al.*, 2009 ▸). *GaussView 5.0* was used to generate the optimized mol­ecular structure (Fig. 6 ▸). The optimized bond parameters obtained are in good correlation with those obtained from SCXRD analysis (Table 2 ▸). The small deviations observed may be attributed to the gas phase (theoretical calculations) compared to the solid phase of SCXRD analysis. The calculated energies of the frontier mol­ecular orbitals are −6.2700 eV and −2.1769 eV. The energy gap Δ*E* was found to be 4.0931 eV (Fig. 7 ▸). The reactivity descriptors calculated from the energy gap value, ionization energy (*I*), electron affinity (*A*), electronegativity (χ), chemical hardness (η), chemical potential (μ), electrophilicity index (ω) and chemical softness (*S*) are 6.2700, 2.1769, 4.2234, 2.0465, −4.2234, 4.3580 eV and 0.2440 eV^−1^ respectively.

**Figure 8:**
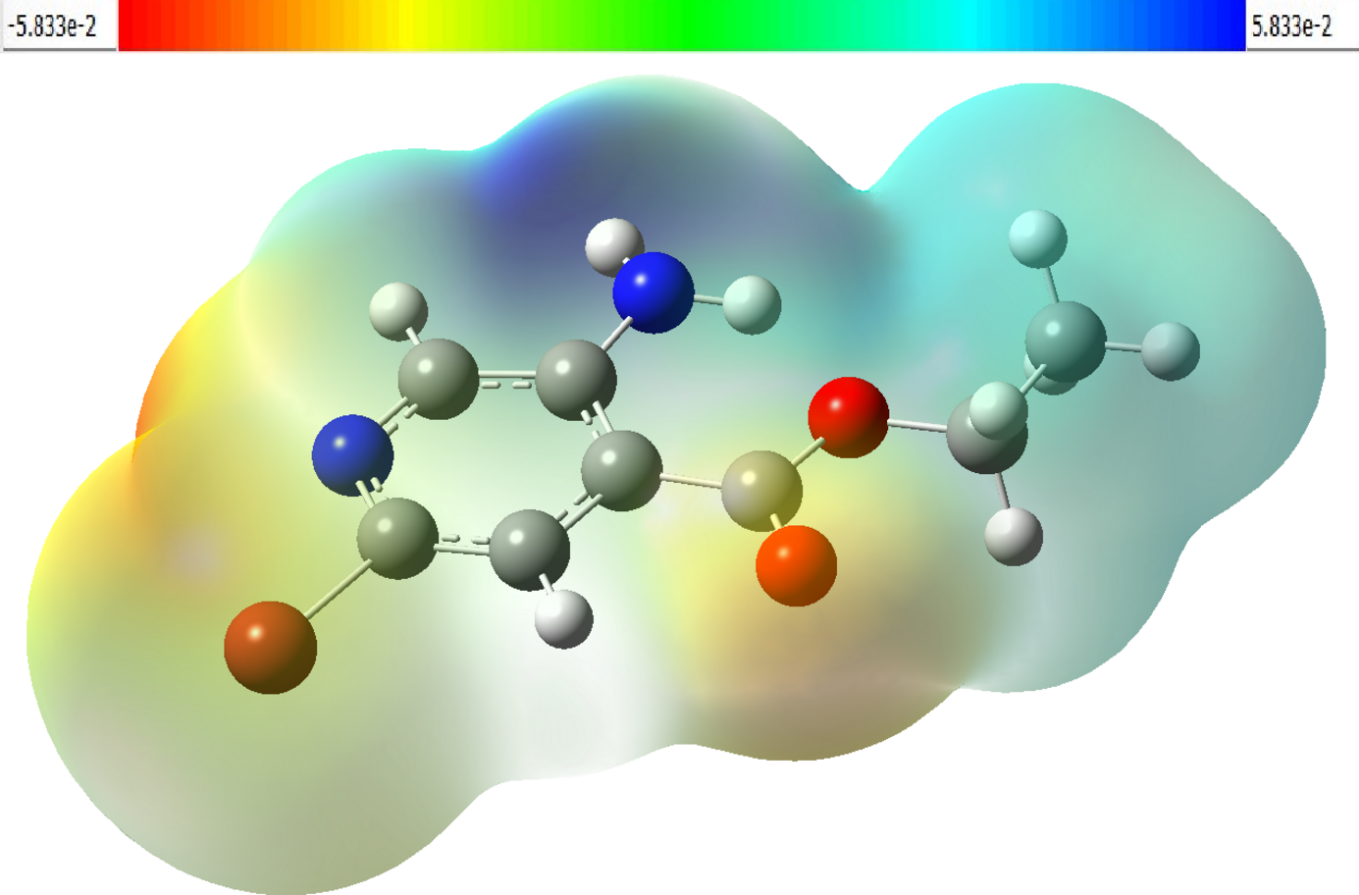
The MEP surface of the optimized mol­ecular structure of the title compound.

The MEP surface of the optimized structure of the title compound is depicted in Fig. 8 ▸. Nucleophilic and electrophilic reactive sites of the mol­ecule are represented by red- and blue-colored regions on the MEP surface. In the MEP surface of the title compound, the pale red color covering the oxygen and nitro­gen atoms of the isonicotinate fragment and the pale-blue color over the amino group are active sites for nucleophilic and electrophilic attack, respectively.

## 7. Mol­ecular docking studies

**Figure 9:**
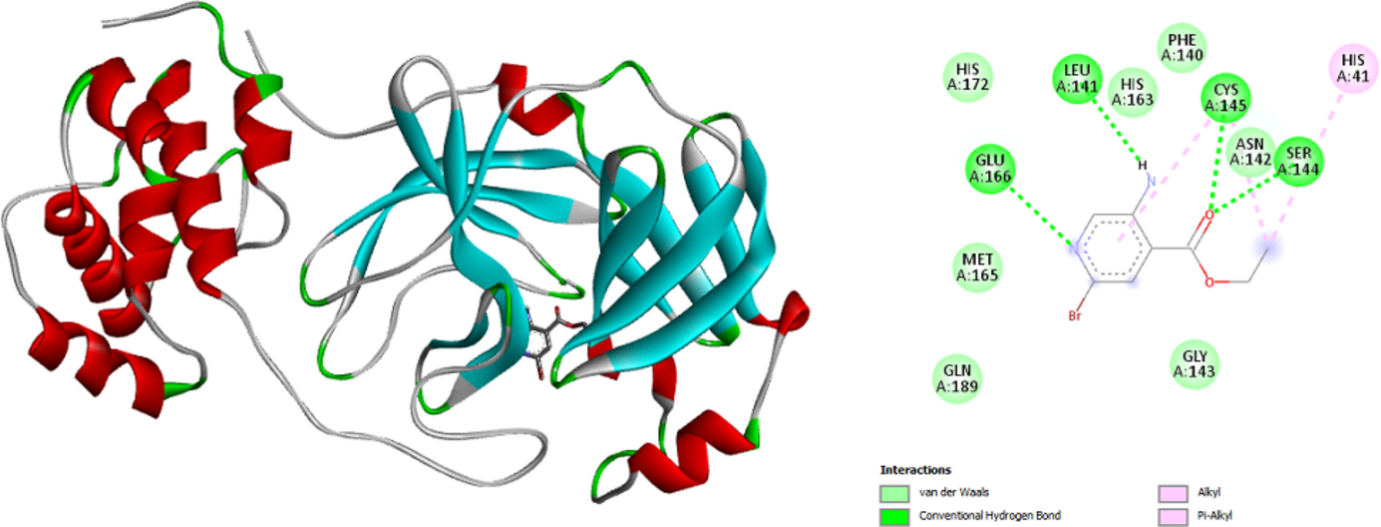
A graphical view of the three-dimensional and two-dimensional docking between the ligand and the receptor protein (covid-19 main protease).

The inter­action of the ligand with the target receptor, covid-19 main protease (PDB ID: 6LU7) was performed using *Autodock Vina 4.2* (Morris *et al.*, 2009 ▸) software. *Biovia Discovery Studio* (Biovia, 2017 ▸) was used for visualizing the inter­actions present between ligand and receptor. The docking results of the ligand with the receptor protein reveal that the ligand has a good binding affinity of −5.4 kcal mol^−1^ and the 2D inter­action view shows conventional hydrogen bonding of GLU A:166, LEU A:141, CYS A:145 and SER A:144 with nitro­gen and oxygen atoms, van der Waals inter­actions between the HIS A:163, ASN A:142 amino residues and ethyl −5-amino −2-bromo­isonicotinate, Fig. 9 ▸. The binding affinity of the title compound with the receptor protein (covid-19 main protease) suggests it to be a potential candidate for pharmaceutical applications. Meanwhile, we have gone through the literature in order to study the efficiency of the title ligand. The docking results of imidazole-anchored azo-imidazole derivatives with the 6LU7 receptor also exhibit a binding affinity of −5.4 kcal mol^−1^ (Chhetri *et al.*, 2021 ▸)

## 8. Synthesis and crystallization

To a stirred a solution of ethyl 3-amino­isonicotinate (800 mg, 1.0 eq) in DMF (8 mL), *N*-bromo­succinimide (NBS; 0.937 mg. 1.1 eq) was added, and the reaction mixture was stirred at room temperature for 6 h. The reaction was monitored by TLC (30% EA: hexa­ne) and it confirmed that the reaction was complete. The reaction mixture was then quenched with water and extracted into ethyl acetate. The organic layer was separated and concentrated to obtain the crude product and purified through Combi-Flash chromatography using 30% EA. Hexane–ethyl acetate was used as mobile phase to obtain the pure compound as a pale-yellow crystal, yield: 98%. A suitable single crystal was used to collect the X-ray data.^1^H NMR (500 Hz) in CDCl_3_, δ 7.96 (*s*, 1H, Ar-H), 7.78 (*s*, 1H, Ar-H), 5.69 (*s*, 2H, NH_2_), 4.36 (*t*, *J* = 7 Hz, 2H, OCH_2_^−^), 1.42 (*t*, *J* = 7 Hz, 3H, –CH_3_) ppm. ^13^C NMR, 125 Hz: δ 165.8, 144.1, 140.3, 126.9, 118.8, 61.6, 14.2 ppm.
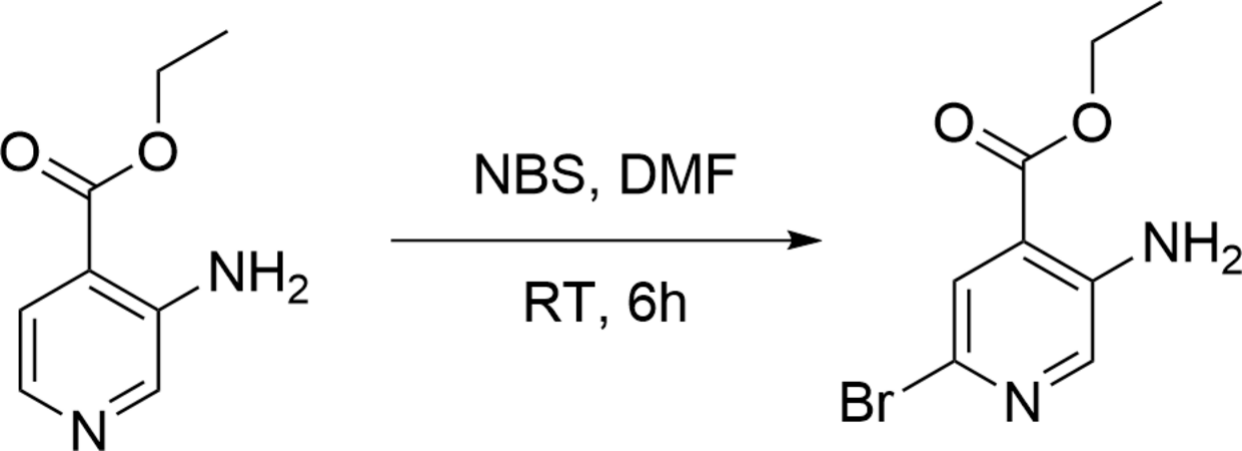


## 9. Refinement

Crystal data, data collection and structure refinement details are summarized in Table 3 ▸. The hydrogen atoms attached to N were located in difference maps. The distances H2*A*/H2*B*—N2 were restrained to 0.82 (2) Å. All other H atoms were positioned with idealized geometry and refined using a riding model with C—H = 0.93–0.97 Å and *U*_iso_(H) = 1.2*U*_eq_(C) or 1.5*U*_eq_(methyl C).

## Supplementary Material

Crystal structure: contains datablock(s) I. DOI: 10.1107/S2056989024010594/oi2013sup1.cif

Structure factors: contains datablock(s) I. DOI: 10.1107/S2056989024010594/oi2013Isup2.hkl

Supporting information file. DOI: 10.1107/S2056989024010594/oi2013Isup3.cml

CCDC reference: 2395555

Additional supporting information:  crystallographic information; 3D view; checkCIF report

## Figures and Tables

**Table 1:** Hydrogen-bond geometry (Å, °)

*D*—H⋯*A*	*D*—H	H⋯*A*	*D*⋯*A*	*D*—H⋯*A*
N2—H2*B*⋯O2	0.87 (3)	2.07 (3)	2.746 (3)	133 (2)
C5—H5⋯O1	0.93	2.37	2.694 (3)	100 (1)
N2—H2*A*⋯N1^i^	0.82 (3)	2.30 (3)	3.096 (3)	166 (3)

Symmetry code: (i) 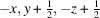.

**Table 2:** Selected bond lengths, angles and torsion angles (Å, °)

Parameter	SCXRD	DFT
Br1—C4	1.903 (2)	1.9253
O1—C6	1.335 (3)	1.3573
O1—C7	1.454 (3)	1.4551
N2—C2	1.354 (3)	1.3704
C3—N1	1.318 (3)	1.3228
N1—C4	1.327 (3)	1.3226
C6—O1—C7	117.00 (2)	116.13
N2—C2—C1	124.5 (2)	124.95
N2—C2—C3	119.3 (2)	118.77
C2—C3—N1	125.1 (2)	124.62
Br1—C4—N1	116.65 (15)	117.07
Br1—C4—C5	119.19 (16)	119.44
O1—C6—O2	123.0 (2)	122.35
C7—O1—C6—O2	−0.6 (4)	−0.34
C7—O1—C6—C1	180.0 (2)	179.93
C3—N1—C4—Br1	178.8 (2)	179.84
N2—C2—C3—N1	−179.0 (3)	−177.72

**Table 3:** Experimental details

Crystal data	
Chemical formula	C_8_H_9_BrN_2_O_2_
*M*_r_	245.08
Crystal system, space group	Monoclinic, *P*2_1_/*c*
Temperature (K)	567
*a*, *b*, *c* (Å)	4.1538 (9), 8.9978 (16), 25.487 (5)
β (°)	92.468 (7)
*V* (Å^3^)	951.7 (3)
*Z*	4
Radiation type	Mo *K*α
μ (mm^−1^)	4.29
Crystal size (mm)	0.32 × 0.27 × 0.21

Data collection	
Diffractometer	Bruker SMART APEXII CCD
Absorption correction	Multi-scan (*SADABS*; Krause *et al.*, 2015 ▸)
*T*_min_, *T*_max_	0.225, 0.401
No. of measured, independent and observed [*I* > 2σ(*I*)] reflections	21643, 2353, 1910
*R*_int_	0.037
(sin θ/λ)_max_ (Å^−1^)	0.666

Refinement	
*R*[*F*^2^ > 2σ(*F*^2^)], *wR*(*F*^2^), *S*	0.032, 0.069, 1.04
No. of reflections	2353
No. of parameters	126
H-atom treatment	H atoms treated by a mixture of independent and constrained refinement
Δρ_max_, Δρ_min_ (e Å^−3^)	0.53, −0.45

Computer programs: *APEX2* and *SAINT* (Bruker, 2014 ▸), *SHELXT2018/2* (Sheldrick, 2015*a* ▸), *SHELXL2019/2* (Sheldrick, 2015*b* ▸) and *Mercury* (Macrae *et al.*, 2020 ▸).

## supplementary crystallographic information

### Ethyl 5-amino-2-bromopyridine-4-carboxylate Crystal data

**Table:**

C_8_H_9_BrN_2_O_2_	*F*(000) = 488
*M**_r_* = 245.08	*D*_x_ = 1.710 Mg m^−^^3^
Monoclinic, *P*2_1_/*c*	Mo *K*α radiation, λ = 0.71073 Å
*a* = 4.1538 (9) Å	Cell parameters from 2367 reflections
*b* = 8.9978 (16) Å	θ = 2.5–29.0°
*c* = 25.487 (5) Å	µ = 4.29 mm^−^^1^
β = 92.468 (7)°	*T* = 567 K
*V* = 951.7 (3) Å^3^	Prism, pale yellow
*Z* = 4	0.32 × 0.27 × 0.21 mm

### Ethyl 5-amino-2-bromopyridine-4-carboxylate Data collection

**Table:**

Bruker SMART APEXII CCD diffractometer	2353 independent reflections
Radiation source: fine-focus sealed tube	1910 reflections with *I* > 2σ(*I*)
Graphite monochromator	*R*_int_ = 0.037
Detector resolution: 0.97 pixels mm^-1^	θ_max_ = 28.3°, θ_min_ = 2.8°
φ and Ω scans	*h* = −5→5
Absorption correction: multi-scan (SADABS; Krause *et al.*, 2015)	*k* = −11→11
*T*_min_ = 0.225, *T*_max_ = 0.401	*l* = −33→33
21643 measured reflections	

### Ethyl 5-amino-2-bromopyridine-4-carboxylate Refinement

**Table:**

Refinement on *F*^2^	Primary atom site location: structure-invariant direct methods
Least-squares matrix: full	Secondary atom site location: difference Fourier map
*R*[*F*^2^ > 2σ(*F*^2^)] = 0.032	Hydrogen site location: mixed
*wR*(*F*^2^) = 0.069	H atoms treated by a mixture of independent and constrained refinement
*S* = 1.04	*w* = 1/[σ^2^(*F*_o_^2^) + (0.0216*P*)^2^ + 0.6676*P*] where *P* = (*F*_o_^2^ + 2*F*_c_^2^)/3
2353 reflections	(Δ/σ)_max_ = 0.001
126 parameters	Δρ_max_ = 0.53 e Å^−^^3^
0 restraints	Δρ_min_ = −0.45 e Å^−^^3^
0.12 constraints	

### Ethyl 5-amino-2-bromopyridine-4-carboxylate Special details

**Table:**

Geometry. All esds (except the esd in the dihedral angle between two l.s. planes) are estimated using the full covariance matrix. The cell esds are taken into account individually in the estimation of esds in distances, angles and torsion angles; correlations between esds in cell parameters are only used when they are defined by crystal symmetry. An approximate (isotropic) treatment of cell esds is used for estimating esds involving l.s. planes.

### Ethyl 5-amino-2-bromopyridine-4-carboxylate Fractional atomic coordinates and isotropic or equivalent isotropic displacement parameters (Å^2^)

**Table:**

	*x*	*y*	*z*	*U*_iso_*/*U*_eq_	
Br1	0.50981 (7)	0.23668 (3)	0.33291 (2)	0.06255 (12)	
O1	0.6756 (4)	0.71953 (17)	0.44789 (6)	0.0519 (4)	
O2	0.4245 (5)	0.91607 (19)	0.41176 (7)	0.0678 (5)	
N2	0.0811 (7)	0.8705 (3)	0.31867 (10)	0.0679 (7)	
C1	0.3910 (5)	0.6820 (2)	0.36780 (8)	0.0389 (4)	
C2	0.1949 (6)	0.7303 (2)	0.32486 (8)	0.0451 (5)	
C3	0.1106 (6)	0.6220 (3)	0.28677 (9)	0.0511 (6)	
H3	−0.019460	0.651860	0.258076	0.061*	
N1	0.2016 (5)	0.4817 (2)	0.28864 (7)	0.0480 (5)	
C4	0.3867 (5)	0.4403 (2)	0.32981 (8)	0.0419 (5)	
C5	0.4870 (5)	0.5336 (2)	0.36960 (8)	0.0414 (5)	
H5	0.617469	0.498569	0.397470	0.050*	
C6	0.4947 (6)	0.7863 (3)	0.41030 (8)	0.0455 (5)	
C7	0.7917 (7)	0.8112 (3)	0.49174 (9)	0.0558 (6)	
H7A	0.612483	0.860208	0.507725	0.067*	
H7B	0.938171	0.886598	0.479721	0.067*	
C8	0.9601 (8)	0.7121 (4)	0.53018 (11)	0.0737 (8)	
H8A	1.039906	0.769478	0.559719	0.111*	
H8B	0.812758	0.638136	0.541835	0.111*	
H8C	1.137029	0.664411	0.513954	0.111*	
H2A	−0.018 (6)	0.889 (3)	0.2911 (12)	0.064 (8)*	
H2B	0.141 (7)	0.934 (3)	0.3432 (12)	0.067 (9)*	

### Ethyl 5-amino-2-bromopyridine-4-carboxylate Atomic displacement parameters (Å^2^)

**Table:**

	*U*^11^	*U*^22^	*U*^33^	*U*^12^	*U*^13^	*U*^23^
Br1	0.07039 (19)	0.04411 (15)	0.0714 (2)	0.00444 (12)	−0.01747 (13)	−0.01644 (12)
O1	0.0718 (11)	0.0421 (8)	0.0399 (8)	0.0006 (7)	−0.0200 (7)	−0.0075 (7)
O2	0.1044 (15)	0.0396 (9)	0.0572 (11)	0.0078 (9)	−0.0225 (10)	−0.0068 (8)
N2	0.102 (2)	0.0467 (12)	0.0519 (14)	0.0056 (12)	−0.0313 (13)	0.0091 (10)
C1	0.0475 (12)	0.0382 (10)	0.0304 (10)	−0.0056 (9)	−0.0048 (9)	0.0015 (8)
C2	0.0559 (13)	0.0426 (11)	0.0359 (11)	−0.0058 (10)	−0.0073 (9)	0.0083 (9)
C3	0.0625 (15)	0.0560 (14)	0.0334 (11)	−0.0075 (11)	−0.0139 (10)	0.0050 (10)
N1	0.0569 (12)	0.0513 (11)	0.0349 (9)	−0.0085 (9)	−0.0090 (8)	−0.0041 (8)
C4	0.0460 (12)	0.0399 (11)	0.0394 (11)	−0.0056 (9)	−0.0026 (9)	−0.0042 (9)
C5	0.0488 (12)	0.0414 (11)	0.0331 (10)	−0.0033 (9)	−0.0090 (9)	0.0005 (8)
C6	0.0589 (14)	0.0401 (11)	0.0367 (11)	−0.0036 (10)	−0.0069 (10)	−0.0005 (8)
C7	0.0683 (16)	0.0557 (14)	0.0418 (12)	−0.0052 (12)	−0.0159 (11)	−0.0167 (11)
C8	0.080 (2)	0.091 (2)	0.0478 (15)	−0.0032 (17)	−0.0203 (14)	−0.0065 (14)

### Ethyl 5-amino-2-bromopyridine-4-carboxylate Geometric parameters (Å, º)

**Table:**

Br1—C4	1.903 (2)	C3—N1	1.318 (3)
O1—C6	1.335 (3)	C3—H3	0.9300
O1—C7	1.454 (2)	N1—C4	1.327 (3)
O2—C6	1.204 (3)	C4—C5	1.368 (3)
N2—C2	1.354 (3)	C5—H5	0.9300
N2—H2A	0.82 (3)	C7—C8	1.478 (4)
N2—H2B	0.87 (3)	C7—H7A	0.9700
C1—C5	1.393 (3)	C7—H7B	0.9700
C1—C2	1.405 (3)	C8—H8A	0.9600
C1—C6	1.483 (3)	C8—H8B	0.9600
C2—C3	1.409 (3)	C8—H8C	0.9600
			
C6—O1—C7	117.00 (18)	C1—C5—H5	120.5
C2—N2—H2A	117 (2)	O2—C6—O1	123.0 (2)
C2—N2—H2B	115.9 (19)	O2—C6—O1	123.0 (2)
H2A—N2—H2B	127 (3)	O2—C6—C1	125.0 (2)
C5—C1—C2	118.54 (19)	O2—C6—C1	125.0 (2)
C5—C1—C6	120.56 (19)	O1—C6—C1	112.08 (19)
C2—C1—C6	120.9 (2)	O1—C7—C8	107.4 (2)
N2—C2—C1	124.5 (2)	O1—C7—H7A	110.2
N2—C2—C3	119.3 (2)	C8—C7—H7A	110.2
C1—C2—C3	116.2 (2)	O1—C7—H7B	110.2
N1—C3—C2	125.1 (2)	C8—C7—H7B	110.2
N1—C3—H3	117.4	H7A—C7—H7B	108.5
C2—C3—H3	117.4	C7—C8—H8A	109.5
C3—N1—C4	116.92 (19)	C7—C8—H8B	109.5
N1—C4—C5	124.2 (2)	H8A—C8—H8B	109.5
N1—C4—Br1	116.65 (15)	C7—C8—H8C	109.5
C5—C4—Br1	119.19 (16)	H8A—C8—H8C	109.5
C4—C5—C1	119.1 (2)	H8B—C8—H8C	109.5
C4—C5—H5	120.5		
			
C5—C1—C2—N2	179.0 (2)	C6—C1—C5—C4	179.9 (2)
C6—C1—C2—N2	−1.2 (4)	C7—O1—C6—O2	−0.6 (4)
C5—C1—C2—C3	0.2 (3)	C7—O1—C6—O2	−0.6 (4)
C6—C1—C2—C3	−180.0 (2)	C7—O1—C6—C1	180.0 (2)
N2—C2—C3—N1	−179.0 (3)	C5—C1—C6—O2	179.6 (2)
C1—C2—C3—N1	−0.1 (4)	C2—C1—C6—O2	−0.2 (4)
C2—C3—N1—C4	0.1 (4)	C5—C1—C6—O2	179.6 (2)
C3—N1—C4—C5	−0.2 (3)	C2—C1—C6—O2	−0.2 (4)
C3—N1—C4—Br1	178.77 (18)	C5—C1—C6—O1	−1.0 (3)
N1—C4—C5—C1	0.3 (4)	C2—C1—C6—O1	179.1 (2)
Br1—C4—C5—C1	−178.67 (16)	C6—O1—C7—C8	175.2 (2)
C2—C1—C5—C4	−0.2 (3)		

### Ethyl 5-amino-2-bromopyridine-4-carboxylate Hydrogen-bond geometry (Å, º)

**Table:**

*D*—H···*A*	*D*—H	H···*A*	*D*···*A*	*D*—H···*A*
N2—H2*B*···O2	0.87 (3)	2.07 (3)	2.746 (3)	133 (2)
C5—H5···O1	0.93	2.37	2.694 (3)	100 (1)
N2—H2*A*···N1^i^	0.82 (3)	2.30 (3)	3.096 (3)	166 (3)

Symmetry code: (i) −*x*, *y*+1/2, −*z*+1/2.

## References

[bb1] Abu-Youssef, M. A., Dey, R., Gohar, Y., Massoud, A. A. A., Öhrström, L. & Langer, V. (2007). *Inorg. Chem.* **46**, 5893–5903. 10.1021/ic0621594 17602550

[bb2] Biovia (2017). *Discovery Studio Visualizer*. Biovia, SanDiego, CA, USA.

[bb3] Bruker (2014). *APEX2* and *SAINT*. Bruker AXS Inc., Madison, Wisconsin, USA.

[bb4] Chhetri, A., Chettri, S., Rai, P., Mishra, D. K., Sinha, B. & Brahman, D. (2021). *J. Mol. Struct.* **1225**, 129230. 10.1016/j.molstruc.2020.129230 PMC7499073 32963413

[bb5] Dardouri, N. E., Hrichi, S., Torres, P., Chaâbane-Banaoues, R., Sorrenti, A., Roisnel, T., Turowska-Tyrk, I., Babba, H., Crusats, J., Moyano, A. & Nasri, H. (2024). *Molecules*, **29**, 3163. 10.3390/molecules29133163 PMC11243641 38999116

[bb6] Frisch, M. J., Trucks, G. W., Schlegel, H. B., Scuseria, G. E., Robb, M. A., Cheeseman, J. R., Scalmani, G., Barone, V., Mennucci, B., Petersson, G. A., Nakatsuji, H., Caricato, M., Li, X., Hratchian, H. P., Izmaylov, A. F., Bloino, J., Zheng, G., Sonnenberg, J. L., Hada, M., Ehara, M., Toyota, K., Fukuda, R., Hasegawa, J., Ishida, M., Nakajima, T., Honda, Y., Kitao, O., Nakai, H., Vreven, T., Montgomery, J. A. Jr, Peralta, J. E., Ogliaro, F., Bearpark, M., Heyd, J. J., Brothers, E., Kudin, K. N., Staroverov, V. N., Kobayashi, R., Normand, J., Raghavachari, K., Rendell, A., Burant, J. C., Iyengar, S. S., Tomasi, J., Cossi, M., Rega, N., Millam, J. M., Klene, M., Knox, J. E., Cross, J. B., Bakken, V., Adamo, C., Jaramillo, J., Gomperts, R., Stratmann, R. E., Yazyev, O., Austin, A. J., Cammi, R., Pomelli, C., Ochterski, J. W., Martin, R. L., Morokuma, K., Zakrzewski, V. G., Voth, G. A., Salvador, P., Dannenberg, J. J., Dapprich, S., Daniels, A. D., Farkas, O., Foresman, J. B., Ortiz, J. V., Cioslowski, J. & Fox, D. J. (2009). *Gaussian 09W*, Revision A. 02. Gaussian, Inc., Wallingford CT, USA.

[bb7] Girgis, A. S., Kalmouch, A. & Ellithey, M. (2006). *Bioorg. Med. Chem.* **14**, 8488–8494. 10.1016/j.bmc.2006.08.041 16973365

[bb8] Groom, C. R., Bruno, I. J., Lightfoot, M. P. & Ward, S. C. (2016). *Acta Cryst.* B**72**, 171–179. 10.1107/S2052520616003954 PMC4822653 27048719

[bb9] Han, X.-J., Zeng, W.-L., Bi, S. & Wan, J. (2007). *Acta Cryst.* E**63**, o1194–o1195.

[bb10] Huras, B., Zakrzewski, J., Krawczyk, M., Bombińska, D., Cieniecka-Rosłonkiewicz, A. & Michalczyk, A. (2017). *Med. Chem. Res.* **26**, 509–517.

[bb11] Krause, L., Herbst-Irmer, R., Sheldrick, G. M. & Stalke, D. (2015). *J. Appl. Cryst.* **48**, 3–10. 10.1107/S1600576714022985 PMC4453166 26089746

[bb12] Li, J., Zeng, W.-L., Wang, M.-H. & Wan, J. (2007). *Acta Cryst.* E**63**, o4177.

[bb13] Liu, K. G., Cai, X. Q., Li, X. C., Qin, D. A. & Hu, M. L. (2012). *Inorg. Chim. Acta*, **388**, 78–83.

[bb14] Macrae, C. F., Sovago, I., Cottrell, S. J., Galek, P. T. A., McCabe, P., Pidcock, E., Platings, M., Shields, G. P., Stevens, J. S., Towler, M. & Wood, P. A. (2020). *J. Appl. Cryst.* **53**, 226–235. 10.1107/S1600576719014092 PMC6998782 32047413

[bb15] Morris, G. M., Huey, R., Lindstrom, W., Sanner, M. F., Belew, R. K., Goodsell, D. S. & Olson, A. J. (2009). *J. Comput. Chem.* **30**, 27852791. 10.1002/jcc.21256 PMC2760638 19399780

[bb16] Sheldrick, G. M. (2015*a*). *Acta Cryst.* A**71**, 3–8.

[bb17] Sheldrick, G. M. (2015*b*). *Acta Cryst.* C**71**, 3–8.

[bb18] Turner, M. J., MacKinnon, J. J., Wolff, S. K., Grimwood, D. J., Spackman, P. R., Jayatilaka, D. & Spackman, M. A. (2017). *CrystalExplorer17.5.* University of Western Australia. http://crystalexplorer.net.

[bb19] Vieriu, S. M., Someşan, A. A., Silvestru, C., Licarete, E., Banciu, M. & Varga, R. A. (2021). *New J. Chem.* **45**, 1020–1028.

[bb20] Wan, J., Li, F., Zeng, W.-L., Li, J. & Bi, S. (2007). *Acta Cryst.* E**63**, o3989.

[bb21] Wang, W. & Mei, Z.-H. (2009). *Acta Cryst.* E**65**, o357. 10.1107/S1600536809001196 PMC2968212 21581956

